# Clinical analysis of influenza in the neonatal intensive care unit

**DOI:** 10.1186/s13052-024-01742-6

**Published:** 2024-09-18

**Authors:** Canyang Jia, Wanyu Jia, Xiaowen Yi, Shuqin Fu, Yajie Cui, Peng Li, Chunlan Song

**Affiliations:** Henan Province Engineering Research Center of Diagnosis and Treatment of Pediatric Infection and Critical Care, Children’s Hospital Affiliated to Zhengzhou University, Henan Children’s Hospital, Zhengzhou Children’s Hospital, Zhengzhou, 450018 China

**Keywords:** Neonatal, Influenza, Virus

## Abstract

**Background:**

The aim was to investigate the clinical characteristics, treatment and prognosis of neonatal influenza.

**Methods:**

The clinical data of 21 neonates who were diagnosed with influenza and admitted to the neonatal intensive care unit of Henan Provincial Children’s Hospital, China, between January 2023 and January 2024 were retrospectively analyzed.

**Results:**

A total of 21 patients were admitted, including 14 with influenza A and 7 with influenza B. Eighteen of these patients were reported to have been exposed to family members with respiratory symptoms before hospitalization. Among all the patients’ mothers, only 1 received the influenza vaccine during pregnancy. Fifteen newborns had fever, 13 appetite loss, 10 cough, 9 shortness of breath, 9 nasal obstruction, 3 runny nose, 3 vomiting, 2 severe wheezing, 2 choking, 2 diarrhea, 1 bloating, and 1 sputum in the throat. The pulmonary auscultation sounds were coarse in 19 neonates, weak in 2, moist rales were appreciated in 5 and wheezing in 4 of them. The peripheral total white blood cell count was normal in 18 patients and elevated in 3. The C-reactive protein level was normal in all subjects, and the procalcitonin level was elevated in 1. Nineteen patients had pneumonia on chest imaging. All patients were treated with oseltamivir and finally recovered.

**Conclusion:**

Influenza A is the most common type of neonatal influenza. The clinical symptoms are atypical, and fever is the main symptom. Treatment with oseltamivir is safe and effective, and the prognosis is mostly favorable.

## Background

Influenza is an acute respiratory infection. Influenza viruses belong to the Orthomyxoviridae family of RNA viruses and are categorized as types A, B, and C. Influenza A, which can infect many animal species, is the most common subtype, influenza B accounts for 11% of cases during nonepidemic periods, and influenza C causes only mild nasal congestion [1]. Infants and young children are at high risk of influenza virus infection, and the incidence of influenza infection in neonates is relatively low because parents generally attempt to minimize their children’s contact with infected individuals [2]. However, during influenza epidemics, neonates may still be infected with influenza viruses due to their imperfect immune systems and lack of influenza vaccination. They are also more likely to develop severe illness and are highly contagious. Neonatal influenza lacks specific clinical manifestations, has an insidious onset, releases virus for a long time, and is highly infectious. If infected neonates are not identified early and isolated in a timely manner, nosocomial infection outbreaks can easily occur in the neonatal intensive care unit, resulting in serious consequences [3]. It is important to study the clinical characteristics and treatment methods for neonatal influenza, but owing to the low infection rate and small number of cases, relatively little research has been conducted on neonatal influenza. Therefore, this study retrospectively analyzed the clinical data of 21 neonates infected with influenza virus in our hospital to provide additional clinical information and further improve the diagnosis and treatment of neonatal influenza.

## Methods

Neonates who were diagnosed with influenza and admitted to the neonatal intensive care unit of Henan Children’s Hospital, China, between January 2023 and January 2024 were selected as the study subjects. The inclusion criteria for patients were as follows: aged ≤ 28 days and diagnosed according to the 2020 version of the “Expert Consensus on the Diagnosis and Treatment of Influenza in Children” influenza diagnostic criteria. The following criteria were used to confirm influenza: a positive influenza virus nucleic acid test; a positive influenza antigen test; a positive influenza virus culture; and levels of influenza virus-specific IgG antibodies in both acute and convalescent serum 4 or more times greater than normal [4]. The exclusion criteria for patients were as follows: serious underlying diseases such as congenital heart disease, genetic metabolic disease, or blood disease; congenital or acquired immunodeficiency; and incomplete history data.

The following critical conditions were considered to indicate severe infection: respiratory distress and/or an increased respiratory rate (> 60 breaths/min); a neurological abnormality: unresponsiveness, lethargy, agitation, convulsions and so on; severe vomiting and diarrhea, with concomitant dehydration; oliguria: a urinary output < 0.80 mL/(kg·h) or a daily urine volume < 200 mL/m^2^ or the emergence of acute renal failure; comorbid pneumonia; significant worsening of the original underlying disease; and other critical conditions requiring hospitalization, such as myocarditis, liver damage, and hyperbilirubinemia. In addition to the above mentions, any of the following conditions were considered to indicate critical infection: respiratory failure; acute necrotizing encephalopathy; septic shock; multiple organ insufficiency; and other serious clinical conditions requiring strict monitoring, such as acute heart failure or cerebral hernia.

General data, including sex, mode of delivery, feeding patterns, birth weight, age at admission, lenght of hospitalization, season of onset, exposure to family members with respiratory symptoms, vaccination status of the mother during pregnancy, patient symptoms, signs, laboratory indicators, imaging findings, treatments, and outcomes, were collected.

SPSS 25.0 was used to analyze the statistical results. The Kolmogorov‒Smirnov nonparametric test was used to determine whether the sample data followed a normal distribution, and *P* < 0.0.5 was considered statistically significant. ‌Countable data were expressed as rates (%), measurement data that conformed to a normal distribution were expressed as x̄±s, and measurement data that did not conform to a normal distribution were expressed as M (Q1, Q2).

## Results

**Fig. 1:**
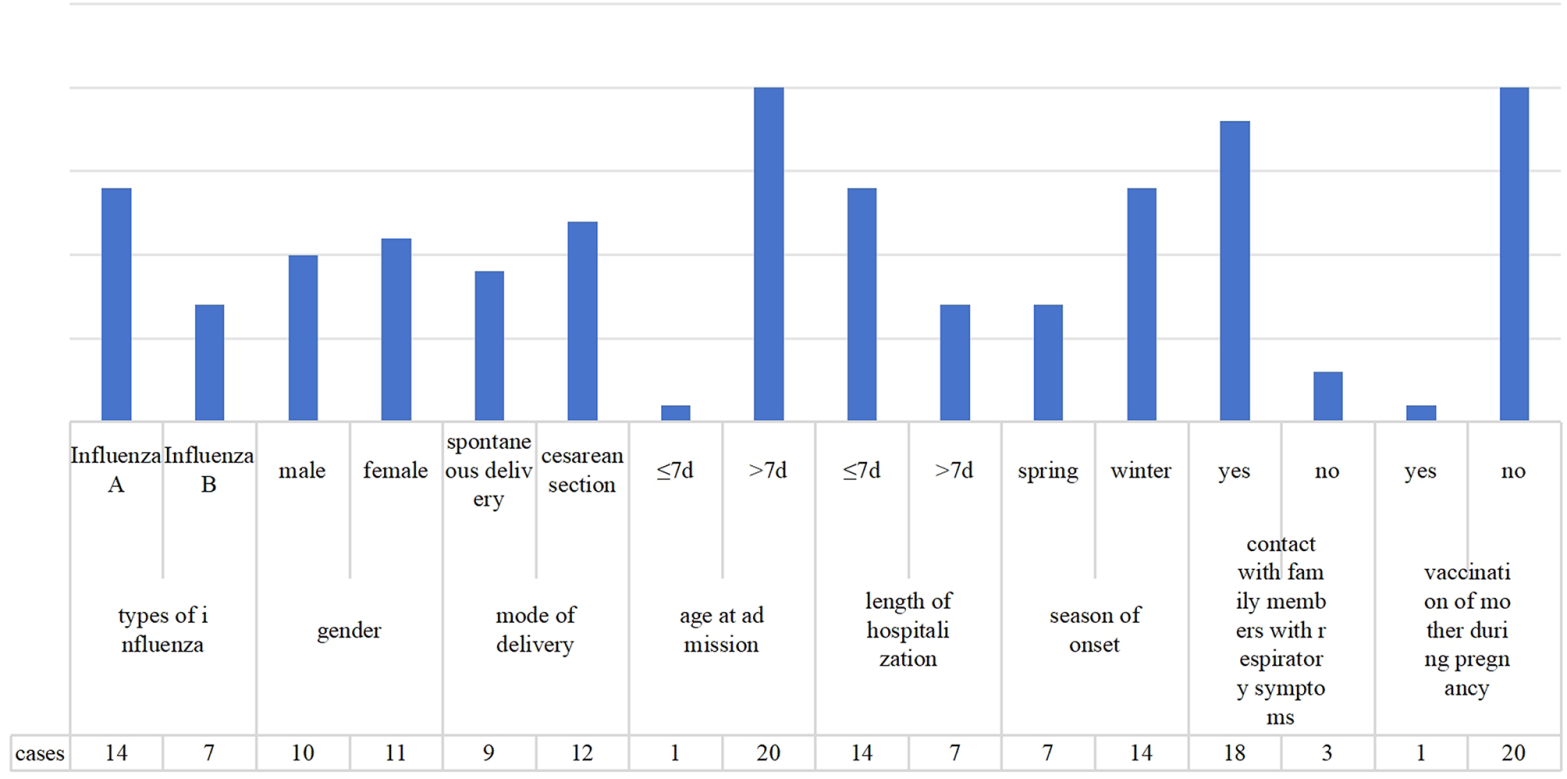
General data of the neonates

According to the inclusion criteria, a total of 21 cases were included in this study, all of whom were full-term infants. A total of 14 patients had influenza A (66.67%), 7 had influenza B (33.33%), 10 were male (47.62%), 11 were female (52.38%), 9 were delivered spontaneously (42.86%), 12 were delivered via cesarean Sect. (57.14%), 10 were exclusively breastfed (47.62%), 8 were formula-fed (38.10%), and 3 received mixed feedings (14.29%). The mean birth weight of these neonates was 3,426.20 ± 343.58 g. The mean age was 18.10 ± 6.66 days, including 1 (4.76%) neonate aged less than 7 days and 20 (95.24%) aged 8 ~ 28 days. The mean lenght of hospitalization was 2 ~ 15 days, in 14 neonates (66.67%) it was ≤ 7 days, and in 7 (33.33%) > 7 days. Seven (33.33%) patients experienced the onset of illness in March, which is during spring in our temperate region. Fourteen (66.67%) patients experienced the onset of illness in winter (6 in January, 6 in November, and 2 in December). Eighteen (85.71%) neonates had a history of contact with family members with respiratory symptoms. Only one mother received the influenza vaccine during pregnancy, and the remaining 20 mothers were not vaccinated, with one mother being unable to be vaccinated due to an allergic reaction to albumen. See Fig. 1.

**Fig. 2:**
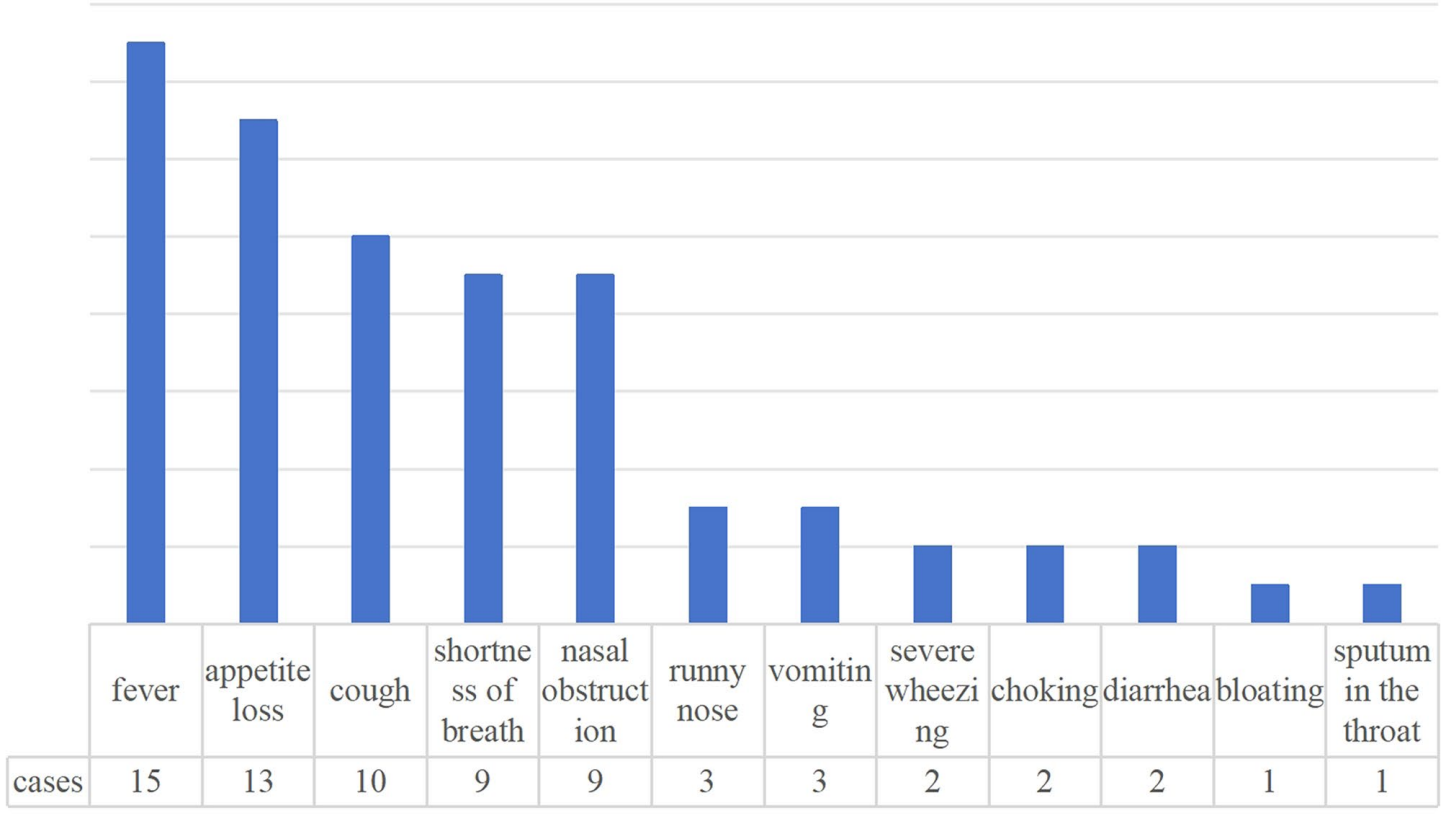
Classification of neonatal influenza symptoms (note that some patients had 2 or more symptoms at the same time)

According to the influenza grading criteria, there were 5 (23.81%) patients in critical condition, including 4 with respiratory failure and 1 with septic shock. The most common symptom of neonatal influenza in our study was fever, which was present in 15 patients (71%). The peak temperature ranged from 37.7 °C to 39.0 °C, in 4 neonates it was ≤ 38.0 °C and in 11 > 38.1 °C. The mean peak temperature was 38.33 ± 0.36 °C. The duration of fever ranged from 0.5 to 5 days, with a mean duration of 1.58 ± 1.12 days. Other symptoms included appetite loss, coughing, shortness of breath, nasal obstruction, runny nose, vomiting, severe wheezing, choking, diarrhea, abdominal distension, and phlegm sounding in the throat. See Fig. 2.

All patients underwent routine blood, C-reactive protein, procalcitonin, and biochemical and blood gas analyses. Routine blood analysis revealed a total white blood cell count of 5.39 ~ 16.95 × 10^9^/L, an elevated leukocyte count (> 15 × 10^9^/L) in 3 patients, and none had a decreased (< 5 × 10^9^/L) leukocyte count. Among these neonates, the percentage of neutrophils ranged from 15.29 to 64.95%, with 6 cases > 50%, and the percentage of lymphocytes ranged from 20.90 to 75.10%, with 10 cases > 50%. The C-reactive protein level was normal (< 10 mg/L) in all individuals. The procalcitonin level was elevated (> 0.5 µg/L) in 1 neonate, and the glutamic oxaloacetic aminotransferase level was elevated (> 40 U/L) in 6. The glutamic protein level was normal (0–40 U/L) in all individuals, the creatine kinase level was elevated (> 200 U/L) in 2, and the creatine kinase isoenzyme level was elevated (> 25 U/L) in 14. Arterial blood gas analysis revealed that the pressure of carbon dioxide was elevated (> 50 mmHg) in 1 neonate.

Six neonates were infected with other pathogens, and 2 of them were infected with more than one pathogen. After the combined respiratory viral nucleic acid test, 4 neonates were found to have viral infections (3 with respiratory syncytial viruses, 1 with human rhinovirus), 3 were found to have bacterial infections (*B. pertussis*, *Hemophilus influenzae*, and multidrug-resistant *Escherichia coli*), and 1 had *Mycoplasma pneumoniae* infection. All neonates [21] had negative blood cultures.

Twenty neonates underwent chest radiography, and one underwent a lung CT scan. All of the individuals had coarse textures in bilateral lung, 16 had patchy shadows, and 3 had small shadows in lung.

All neonates were treated with the neuraminidase inhibitor anti-influenza virusoral or oseltamivir 3 mg/(kg·d) for 5 days, and none experienced adverse drug reactions (such as rash, liver or kidney damage, or convulsions). There were no patients receiving intravenous peramivir. Eighteen patients were treated with additional antibiotics (ceftazidime in 8, cefotaxime sodium in 5, cefoperazone-sulbactam in 4, and azithromycin in 2). One neonate was treated with a combination of two antibiotics (cefoperazone sulbactam with azithromycin).

Seven neonates underwent respiratory support, all of whom received noninvasive breathing, including high-flow nasal cannula therapy in 4 and nasal intermittent positive pressure ventilation in 3. Eleven neonates were treated with cough suppressants/nebulizers, and 3 were treated with glucocorticosteroids (methylprednisolone in 2and dexamethasone in 1). Three patients in critical condition were treated with vasoactive medication (dobutamine), and 4 in critical condition were treated with supportive therapy with human gammaglobulin.

All 21 neonates were cured after treatment, and the prognosis was good according to telephone follow-up until one month after discharge.

## Discussion

In this study, 66.67% of the neonates were infected with influenza A. This finding shows that neonates are predominantly infected with such viral type, which is consistent with findings in children of other ages [5]. In this study, 95.24% of the patients were more than 8 days, suggesting that the influenza virus is more likely to be detected in patients with increased exposure to external environmental factors and a 1 ~ 4-day incubation period after influenza infection. The seasons of onset in this study were all spring and winter for all individuals, which is consistent with the characteristics of influenza epidemics in temperate zones [6, 7].

Neonatal influenza is caused mainly by intrapartum and postpartum horizontal contagion and rarely occurs through vertical transmission [8]. Contact with family members with respiratory symptoms is the most common route of transmission of neonatal influenza [9]. As pregnant women are in a state of immunosuppression and belong to the influenza-susceptible population, influenza vaccination can not only effectively reduce the risk of maternal infection but also transfer protective antibodies to the fetus through the placenta, which can help to protect neonates against influenza virus [10–12]. However, the current influenza vaccination rate for pregnant women in China is 1.5% [13], and the U.S. 2019 ~ 2020 Epidemic Season Survey reported that the influenza vaccination rate for pregnant women was 61.2% [14]. The significantly lower vaccination rate in China is due mainly to concerns about the safety of the influenza vaccine during pregnancy and insufficient awareness of the hazards of influenza infection in pregnant women [15]. Recent studies have shown that influenza vaccination in pregnant women does not increase the risk of intrauterine infections, gestational hypertension, gestational diabetes mellitus, preeclampsia, or other pregnancy complications [16]. It has not been shown to increase the risk of adverse pregnancy outcomes such as preterm labor, congenital malformations [17–20] or fetal death [21, 22]. Moreover, influenza vaccination can reduce the risk of stillbirths, small-for-gestational-age babies, and preterm births associated with maternal influenza infection [8]. The Chinese Center for Disease Control and Prevention recommends pregnant women as a priority population for influenza vaccination, which can be administered at any stage of pregnancy [10]. Public education about influenza vaccination for pregnant women should be strengthened, and pregnant women should be actively encouraged to receive influenza vaccination.

Ten neonates were exclusively breastfed. Infants are vulnerable to infection because of their immature immune systems. Mothers can transfer immune cells during breastfeeding to provide passive immunity to their infants. T cells derived from human milk may provide additional protection to neonates until their T cells develop and mature [23]. In addition, exclusively breastfed infants whose mothers received the influenza vaccine while pregnant had the lowest incidence of respiratory illness with fever [24]. Compared with those who did not receive the influenza vaccine, women who received the influenza vaccine had greater numbers of genes encoding T-cell surface markers (CD44, CD8A, CD62L and CD25) in breast milk [25]. Maternal influenza immunization can reduce respiratory disease in breastfeeding infants through human milk antibodies produced by plasma cells [24]. Exclusive breastfeeding should be encouraged by increased efforts [26].

In our study, the leukocyte count, C-reactive protein level, and procalcitonin level were mostly normal after influenza infection in neonates. Some of the patients had increased glutamic oxaloacetic aminotransferase, creatine kinase, and creatine kinase isoenzyme levels. Lobular changes due to pneumonia were observed during imaging, which was similar to what has been observed in children of other ages [5]. However, studies have reported that the leukocyte classification after influenza infection in children is predominantly neutrophilic [5]. In our study, the leukocyte classification was predominantly lymphocytic, which is considered to be because the characteristics of leukocyte classification in neonates are different from those in children of other ages.

There were 6 neonates infected with other pathogens in our study, 4 of whom were in critical condition. The combined pathogens included respiratory syncytial virus, human rhinovirus, *Mycoplasma pneumoniae*, *Hemophilus influenzae*, multiresistant *Escherichia coli*, and *B. pertussis*. After the COVID-19 pandemic, the resurgence of *B. pertussis* has become a significant public health challenge worldwide. Pertussis is a highly contagious severe acute respiratory disease that is especially dangerous for infants and may cause neonatal leukocytosis, pulmonary hypertension, and hypoxemia. Infants under six weeks of age with pulmonary hypertension are at the highest risk of death [27]. Since the symptoms of pertussis overlap with those of acute respiratory viral infections, prompt testing for *B. pertussis* should be performed to avoid delayed diagnosis [27, 28]. In addition, 2 patients in critical condition in our study were infected with more than 2 pathogens. The results suggest that patients in critical condition may have mixed infections, and respiratory pathogen testing and sputum culture should be performed in a timely manner when patients have rapid progression and recurrent disease.

We observed that fever was the main symptom of neonatal influenza, which was consistent with the findings of previous reports [2, 29], with fever peaks ranging from 37.7 °C to 39.0 °C. The fever duration ranged from 0.5 to 5 days, which was shorter than that of children in other age groups [30] and was considered to be related to neonatal temperature characteristics, multiple heat dissipation pathways, and rapid heat dissipation [31]. There were 4 neonates who had only fever and appetite loss without respiratory symptoms, which was difficult to distinguish from the manifestations of neonatal sepsis. In our study, six neonates with influenza had only cough or nasal congestion without fever as the main manifestation. Therefore, neonatal influenza has no specific clinical manifestations, and distinguishing influenza from other respiratory pathogen infections on the basis of symptoms alone is difficult. There were 4 neonates with vomiting or diarrhea, among whom 3 had influenza B. These data seem to indicate that digestive symptoms occur mainly in influenza B patients. However, the sample size was too small to obtain conclusive results, and we will consider expanding the sample size for statistical comparisons of gastrointestinal symptoms between neonates with influenza A and those with influenza B.

Neonatal influenza treatment involves mainly neuraminidase inhibitors [32]. The optimal time for administering oseltamivir is within 48 h of the appearance of influenza symptoms. Ten patients in this study were administered the drug after 48 h of symptom onset, and the treatment was still effective, with no adverse effects. These findings indicate that oseltamivir is safe and effective for the treatment of neonatal influenza and that it is recommended for use even if it is administered more than 48 h after symptom onset. For patients with poor anti-influenza virus therapy efficacy for 3 ~ 5 days or recurring illness, as well as early signs of critical influenza, physicians need to consider the combination of bacterial infections and should provide timely antibiotic treatment [4]. In this study, 18 patients were treated with antibiotics. In addition to the situation described above, all of the neonates with positive bacterial etiology tests or laboratory indices indicating bacterial coinfection were also given antibiotic therapy. Some febrile neonates with suspected sepsis were given antibiotics during early treatment, which were stopped in a timely manner after bacterial infection was excluded. In addition, critical cases were treated in conjunction with dopamine, glucocorticoids, gammaglobulin and other adjuvant therapies. Dopamine can be used as the vasoactive drug of choice for the treatment of septic shock. Low doses of glucocorticoids can reduce airway hyperresponsiveness and pulmonary exudation in patients. Intravenous immunoglobulin can neutralize toxins in the blood circulation, inhibit immune damage and improve host defenses [33]. Acute necrotizing encephalopathy can be treated with a combination of glucocorticoids and immunoglobulin [4].

The prevention of influenza in neonates relies mainly on primary prevention, which involves encouraging neonatal medical staff in the neonatal intensive care unit and family members, especially mothers and older children, to receive the influenza vaccine, which can be recommended by family pediatricians in addition to birthing centers neonatologists [34]. Moreover, promoting breastfeeding, performing droplet and contact isolation for influenza patients [2], and encouraging family members with respiratory symptoms to use masks can prevent viral transmission to neonates. In particular, during influenza epidemic seasons, influenza testing of influenza-like cases in the ward needs to be carried out in a timely manner to isolate patients as early as possible to avoid nosocomial transmission. Influenza vaccination is not recommended in such population because of a lack of research data on its safety and efficacy, combined with neonatal immune characteristics [2, 4]. Oral oseltamivir has be given for prophylaxis in neonates with influenza exposure in some clinical studies [35].

This study has several limitations, mainly due to the small sample size, which can be expanded to further explore the comparative analysis of clinical data between neonates with influenza A and B. In addition, we would like to follow up our patients to determine whether there have long-term impact of influenza virus infection.

## Conclusions

Influenza A is the most common cause of neonatal influenza and is frequently observed in neonates more than 8 days. Neonatal influenza spreads mainly through contact with family members with respiratory symptoms. Fever is the main symptom of neonatal influenza, which has no specific clinical manifestations. The leukocyte count and C-reactive protein level are mostly normal in neonates infected with influenza. During influenza epidemics, timely laboratory tests are needed for early recognition of neonatal influenza patients with fever and/or respiratory symptoms. Oseltamivir-related anti-influenza treatment is safe and effective, and the prognosis is generally good. Strict sterilization and timely isolation are key measures to prevent neonatal influenza virus.

## Data Availability

The datasets analysed during the current study are available from the corresponding author upon reasonable request.To protect study participant privacy, our data cannot be shared openly.
